# Analysis of Hair Cortisol as an Indicator of Chronic Stress in Pigs in Two Different Farrowing Systems

**DOI:** 10.3389/fvets.2021.605078

**Published:** 2021-01-28

**Authors:** Dierck-Hinrich Wiechers, Susanne Brunner, Swetlana Herbrandt, Nicole Kemper, Michaela Fels

**Affiliations:** ^1^Institute for Animal Hygiene, Animal Welfare and Farm Animal Behavior, University of Veterinary Medicine Hannover, Foundation, Hanover, Germany; ^2^Department of Statistics, TU Dortmund University, Dortmund, Germany

**Keywords:** hair cortisol, chronic stress, pig, farrowing pen, hair

## Abstract

Confinement to farrowing crates is known to prevent sows from performing natural behavior, impairing animal welfare and possibly causing chronic stress. Hair cortisol analyses are increasingly used to detect chronic stress in animals. In the present study, hair samples were collected in the neck of sows kept either in farrowing crates (FC, *n* = 31) or in a loose-housing system (LH, *n* = 30) in six batches. Cortisol was extracted and analyzed using chemiluminescence immunoassay. Mean hair cortisol concentrations (HCC) did not differ significantly between the systems (LH: 1.85 ± 0.82 pg/mg, FC: 2.13 ± 1.53 pg/mg, *P* = 0.631). HCC was also not affected by other factors, such as sows' parity, number of piglets, skin lesion score or sow's weight loss during the farrowing period. However, highly significant differences were found in hair growth rates between different regions within the 20 × 30 cm shaving area. While the hair in both lateral parts of the shaving area grew almost identically (left: 7.48 ± 3.52 mm, right: 7.44 ± 3.24 mm, *P* = 1.00), the hair grew more in the area above the spine (12.27 + 3.95 mm, *P* < 0.001). In both systems, the mean individual lesion score of sows declined from the beginning to the end of the housing period (*P* < 0.001). No difference was found between FC and LH sows at any time (*P* > 0.05). Since neither the amount of skin lesions nor HCC differed between LH and FC sows, it may be concluded that confining sows in farrowing crates did not affect chronic stress levels. However, results may be affected by a downregulation of the hypothalamic-pituitary-adrenal axis during long-term stress, resulting in lower cortisol levels over time. HCC in sows may also be influenced by a dominant stressor, such as farrowing or the presence of suckling piglets. Thus, for a comparison of different farrowing systems regarding chronic stress, the use of hair cortisol measurement seems to be limited. The present results revealed that differences in hair growth rate within the same body region exist. This important finding should be considered when collecting hair samples in pigs, since hair cortisol concentrations may vary depending on hair growth and length.

## Introduction

The subject of animal welfare in intensive pig farming has become increasingly important for the public in recent years (1). It is already scientifically recognized that farrowing crates restrict sows, not just in their locomotion but also in other natural behaviors (2), causing stress for the confined animals. Loose housing systems without farrowing crates seem to be advantageous in this regard (3), and thus several are currently being researched to improve animal welfare. To evaluate housing systems concerning animal welfare, specific indicators, which refer mostly to physical impacts and the animals' behavior are used (4). However, a housing system should also be evaluated regarding the level of stress which the animals experience there (5). Thus, studies comparing different housing systems often include measurements of stress levels as well. While the term “stress” was only indirectly addressed in earlier definitions of animal welfare, such as the five freedoms (6), today it is often included in the definition of animal welfare itself. From this point of view, the perception of chronic stress is incompatible with good animal welfare (7).

A widely used method to quantify stress is to measure the cortisol level in body fluids or excreta as a biomarker. Cortisol is the main glucocorticoid in most mammals (8) and is produced and released into the blood by the adrenal glands after a stimulus of adrenocorticotropic hormone (ACTH). This hormone is emitted by the activated hypothalamic-pituitary-adrenal axis (HPA axis) (9) after the animal has been confronted with a stressor. The causes of stress in pigs are multifactorial, and can be categorized as social, environmental, metabolic, immunological, or due to handling (10). Acute stress leads to a rapid increase in glucocorticoids, with a peak after about 15–30 min. It is temporarily conducive to adaption to external threats by the redistribution of energy in the organism. In contrast, in case of chronic stress, a long-term elevated glucocorticoid level can be deleterious in many ways (11). Several matrices can be used for cortisol analysis: blood plasma, saliva, urine, feces, milk, and hair (9, 12). Due to the rapid increase after a stimulus occurs and the equally rapid decrease after its removal, cortisol levels in plasma and saliva are highly variable point samples. Even in urine and feces cortisol represents just a time period of 24 h or less of stress experience, so that none of these matrices provide a long-term view of HPA axis activity (13, 14).

Koren et al. (15) carried out one of the earliest studies on the possibility of hair cortisol measurements in animals. Since then, research on this subject has been increasingly intensified in order to be able to measure this hormone as a chronic parameter of stress (14). In addition to providing the advantage of the long-term analysis, collecting hair is a non-invasive method, and the sampling procedure has no influence on the measured values themselves. In contrast, stressful and painful blood sampling can affect cortisol measurements in blood plasma (13). To explain the storage routes of cortisol into the hair shaft, the multicompartment model is often suggested as a basic hypothesis (16). According to this model, the pathway of cortisol release into the hair occurs not only by diffusion from blood into the follicle during the anagen phase of hair formation, but the glucocorticoid can also be incorporated into the hair by an overlaying film of sweat and sebum of hair-associated glands. A further possible way is by incorporating cortisol into the hair via external substances from the environment, after the hair has grown past the outer skin layer. In this case, it would even be conceivable that cortisol is incorporated after hair sampling, thus contaminating the samples. Furthermore, the glucocorticoid can be synthesized and secreted by the hair follicle itself as a functional equivalent of the HPA axis, caused by local stressors on the skin and hair. Since this reaction is independent of the central HPA axis activity, it can be assumed that an additional “peripheral” stress axis exists, with its own local stress response in addition to the systemic reaction (17, 18).

Considering all these possible origins, the question remains to what extent measured hair cortisol concentrations (HCC) are influenced by systemic cortisol levels and whether they actually reflect the HPA axis activity. Some studies have shown that HCC increased in times of higher plasma cortisol levels, or when ACTH was applied to the organism experimentally (19–21). Thus, the HPA axis-dependent cortisol concentration in the hair seems to be sufficiently high to be able to regard the hair as an appropriate medium for chronic stress detection. Further studies showed a correlation between the concentrations of cortisol in hair and feces (22), urine, serum and saliva (23), and underline the possibility of cortisol analyses in hair to detect chronic stress. However, it should be considered that although hair cortisol seems to have a long-term stability of months or years, cortisol can also escape from the hair due to environmental factors (24).

Even if some doubts remain, and there is a need for further research, detection of hair cortisol is increasingly considered a useful marker to determine chronic stress in animals. Therefore, it may be suitable for assessing long-term stress caused by different housing systems for farm animals. Hence, the aim of the present study was to explore the applicability of hair cortisol measurement to detect chronic stress in sows kept in two different farrowing systems. Moreover, factors which affected the sows' stress levels in the farrowing systems should be analyzed as well. Since physical damage in pigs can also influence chronic stress levels (25), the occurrence of skin lesions in sows was investigated using a lesion score and their impact on HCC was also determined.

## Materials and Methods

### Animals, Housing, and Handling

The study was conducted between June 2018 and January 2019 as part of a larger research project at the research farm of the Lower Saxony Chamber of Agriculture in Wehnen, Bad-Zwischenahn, Germany. The animals were kept in accordance with the European Directive 2008/120/EC and the corresponding German national law (Tierschutzgesetz and Tierschutz-Nutztierhaltungsverordnung). The experiments did not include any invasive procedure involving the animals. The study was reviewed and received approval from the Animal Welfare Officer of the University of Veterinary Medicine Hannover, Hannover, Foundation, Germany.

**Figure 1:**
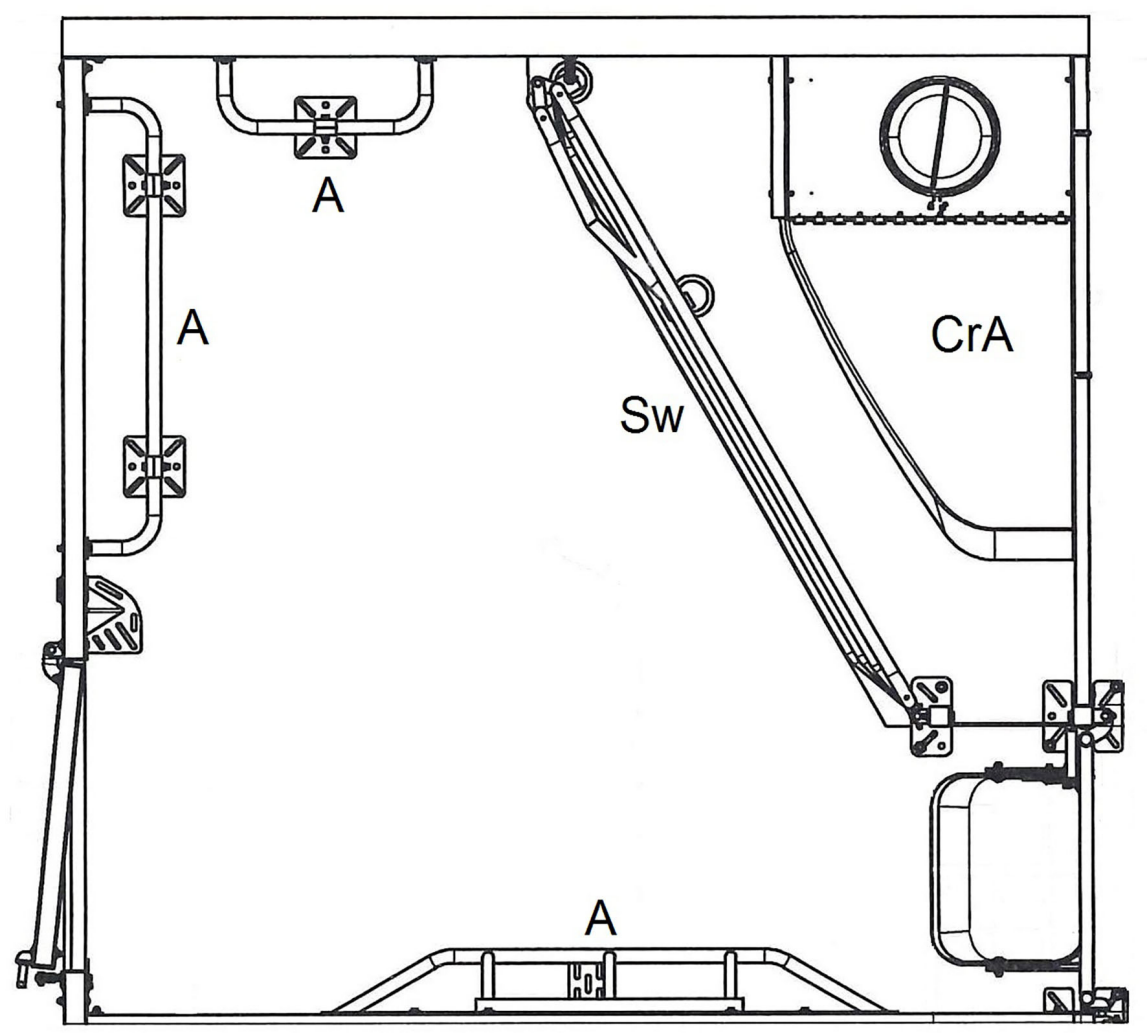
Single loose housing pen (LH). CrA, creep area; Sw, swing gate; A, anti-crushing bars. © Big Dutchman International GmbH, Vechta, Germany.

In the study, two different farrowing systems for sows were compared: conventional pens with farrowing crates (FC) and a loose-housing system without farrowing crates (LH). Both systems were installed in adjacent rooms, and in both systems the sows were single-housed. The LH system had six pens per room and the FC system was equipped with eight pens. Both systems were provided by the same manufacturer (Big Dutchman International GmbH, Vechta, Germany). A single LH pen (Figure 1) was 250 cm long and 240 cm wide (6 m^2^). A space of 4.01 m^2^ was available for the loose-housed sow, separated from the creep area for piglets by a swiveling iron grid, which could be used for confining the sow for different management procedures. The separated two-sided-open creep area (125 × 75 cm) was equipped with a 150 W infrared light heating lamp. The floor of the creep area was covered with a solid rubber mat. To prevent the piglets from being crushed by the sow, anti-crushing bars were installed as a mushroom-shaped protrusion at the long side of the pen. Piglet protection bars were located at the two shorter free sides.

**Figure 2:**
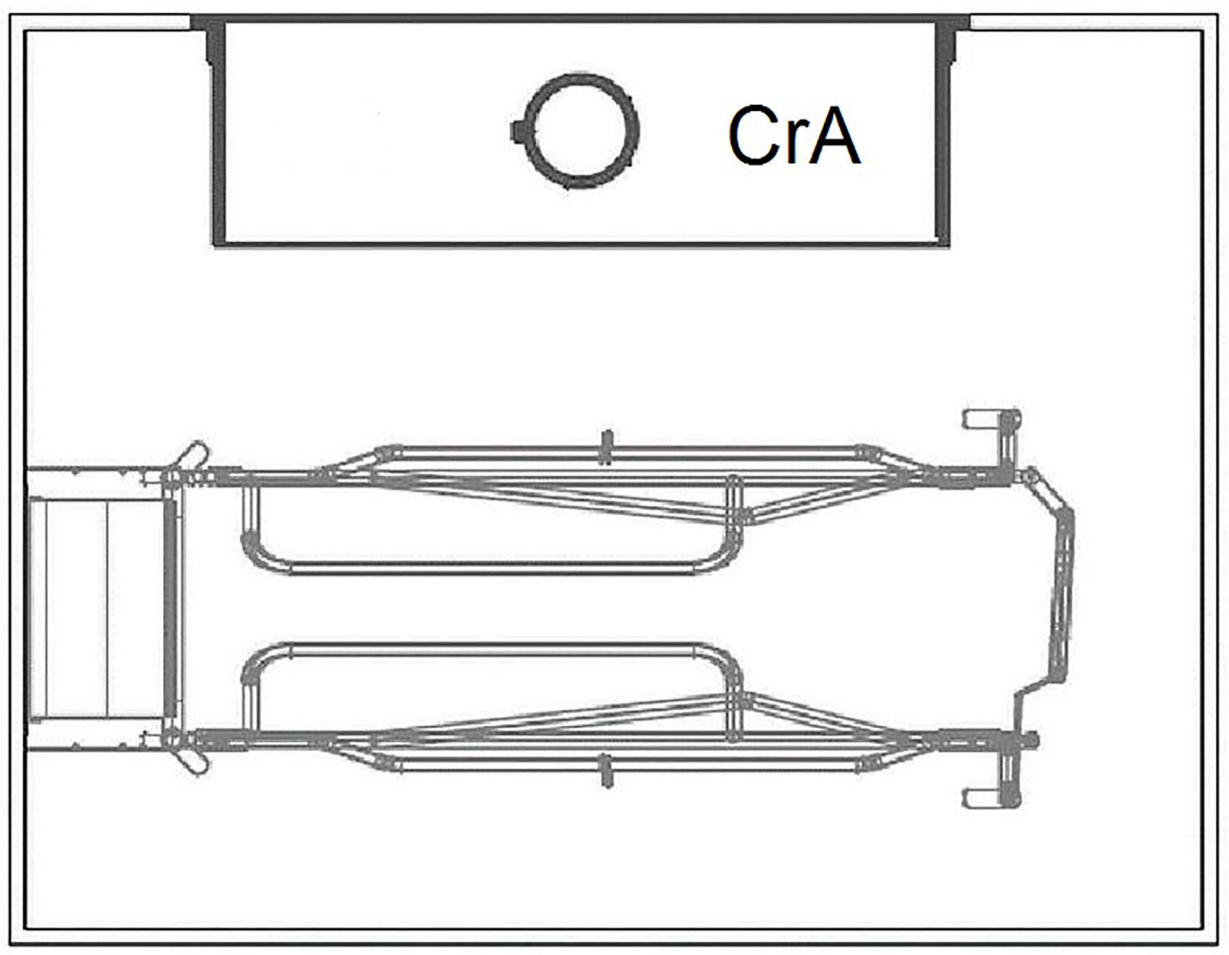
Pen with farrowing crate (FC). CrA, creep area. © Big Dutchman International GmbH, Vechta, Germany.

The FC pen (Figure 2) measured 260 × 200 cm (5.2 m^2^) and included a 190 cm-long and 80 cm-wide (1.52 m^2^ usable area for the sow) farrowing crate in the center of the pen. The creep area for piglets was located parallel to the sow's crate and was open on three sides. It was 150 cm long and 60 cm wide and heated by an infrared lamp as well as by a heatable polymer concrete floor. Both housing systems were equipped with the same slatted plastic flooring (10 mm gaps and 11 mm slats), with a non-perforated lying area for the sow, and were subject to the same management procedures. In the LH pen as well as in the FC pen, sows were offered a jute sack as nest-building material in the period before farrowing. As further manipulable material, cotton ropes were offered - one for the sow and a smaller one for the piglets. In addition, a rack with hay was installed in each LH pen. If necessary, all consumed or worn materials were replaced.

Before entering the farrowing systems, pregnant sows were housed in groups of three to five animals. Five days before the expected farrowing date, sows were moved to the farrowing pens and thus, were single housed either in FC or in LH pens. Six sows were housed in each farrowing system per batch. At the beginning of the study, the sows were randomly assigned to the two housing systems and thereafter were always allocated to the same housing system. Before entering their pens, the sows were washed and weighed using digital scales (82-b2, RHEWA WAAGENFABRIK, August Freudewald GmbH & Co. KG, Mettmann, Germany). The weight of the piglets was individually recorded within 24 h after birth (scale: SC-A, T.E.L.L. Steuerungssysteme GmbH & Co. KG, Vreden, Germany) and an ear tag was immediately applied to identify the individual animals. The teeth (canines) of the piglets were shortened at the same time. To obtain litter sizes that were as homogeneous as possible, cross-fostering was carried out within the same housing system between three and 72 h after birth. After 28 days, piglets were weaned, reweighed individually and then transferred to the farm's own rearing unit.

While LH sows were never confined during the entire housing period (free farrowing), FC sows were permanently fixed in the crate. Feeding-management in the two systems was the same: sows received a commercial lactation diet twice a day (07:30 and 16:30). The amount of feed was rationed on the days before farrowing (maximum 5 kg per day) and on the day of parturition (maximum 2 kg per day). After parturition, the feed amount was increased by about 0.5 kg per day to reach an ad libitum feeding level after about 14 days (8–9 kg per day).

The sows left the farrowing pens after a period of 33 days, were weighed for the second time and entered the service center for the following reproduction cycle.

In both farrowing systems, temperature and air humidity were measured every 2 min in the respective rooms. The sensors (DOL 114 and DOL 12, dol-sensors A/S, Aarhus, Denmark and 135pro, Big Dutchman International GmbH, Vechta, Germany) were placed at the animals' body height in a farrowing pen in the middle of the room.

In a total of six batches, data of 69 sows (Landrace x Large White, db.Vicoria, BHZP GmbH, Dahlenburg, Germany) from first to seventh parity (LH: 3.8 ± 1.8, FC: 4.3 ± 1.8) were obtained. In five batches, data on all recorded parameters were collected for all sows (*n* = 60). In order to increase the number of hair samples for cortisol analyses, an additional batch was added for this purpose. As some sows were sent for slaughter before hair sampling, and hair length measurement was not possible for every animal, the number of sows to be investigated for several parameters was slightly reduced.

### Video Analysis

Cameras (Everfocus ez.HD, Everfocus Electronics Corp., New Taipei City, Taiwan) were installed above each pen to record the animals' behavior. They were arranged at the cable duct, directly above the middle of the pen, to observe the entire area from a top view. The cameras were connected to a digital video recorder (Everfocus ECOR FHD 16 × 1, Everfocus Electronics Corp., New Taipei City, Taiwan), which recorded continuously on hard drives over the entire experimental period. The behavior of 60 sows in five consecutive batches was analyzed regarding the occurrence of stereotypies. The associated ethogram is shown in Table 1. The animals were observed by the same observer for an 8-h period per Saturday - 4 h in the morning (6:00–10:00) and four in the afternoon (13:00–17:00). There were 5 days of observation: one Saturday before farrowing (mean proximity to farrowing: 3.7 ± 1.5 days), and four Saturdays after farrowing (mean proximity to farrowing: 3.2/10.2/17.2 and 24.2 ± 1.5 days). On those observation days, the occurrence of stereotypies was analyzed for all sows during the time frames using the one-zero sampling method (i.e., Yes-No scale). The general occurrence of stereotypies in a sow (at least one Yes during the 40 h study period) was included in the statistical model. The observed frequencies and types of stereotypical behavior were not part of this analysis.

**Table 1:** Overview of the sows' stereotypies analyzed in the present study.

**Stereotypy**	**Definition**
Head waving	Sow moves its head up and down
Bar biting	Sow bites into the bars of the pen
False chewing	Sow chews independently of feed intake, formation of foam at the mouth

The total results of the behavioral analyses are planned to be published in a following paper.

### Lesion Scoring

In five batches, the sows (*n* = 60) were scored individually concerning the occurrence of skin lesions by one trained observer at three different time points per batch. Sows were first scored at the day of entering the farrowing systems to record the injuries that resulted from group housing during pregnancy. The next scorings were performed after 13 and 30 days in both farrowing systems. In accordance with the scoring scheme of Nicolaisen et al. (26) (Table 2), different body regions were assessed for the two body sides of each sow separately: head, ears, shoulder/neck, forelimbs, lateral side, ham, hind limbs, and the udder. For the loin, the sows received just one scoring grade. For each individual, the scores given for different body regions were added up to a cumulative body lesion score (BLS). Scoring results of the two udder sides were added up analogously to a cumulative udder lesion score (ULS).

**Table 2:** Scoring scheme for skin injuries (26).

**Score**	**Definition**
0	No injuries
1	A small number (<5) of superficial scratches
2	A mean number (5–10) of superficial or a small number of deep scratches (<5)
3	A high number (>10) of superficial or a mean up to a high number of deep scratches (>5)

*Superficial injuries were defined as those of the outer skin layers with a minimal reddening or little bleeding at this position. Instead of a blood spot, there could also be a scab. Deep injuries were those that penetrated to the lower skin layers, with reddening, bleeding or scabbing. Furthermore, necrotic or purulent injuries were possible as well*.

### Hair Samples

**Figure 3:**
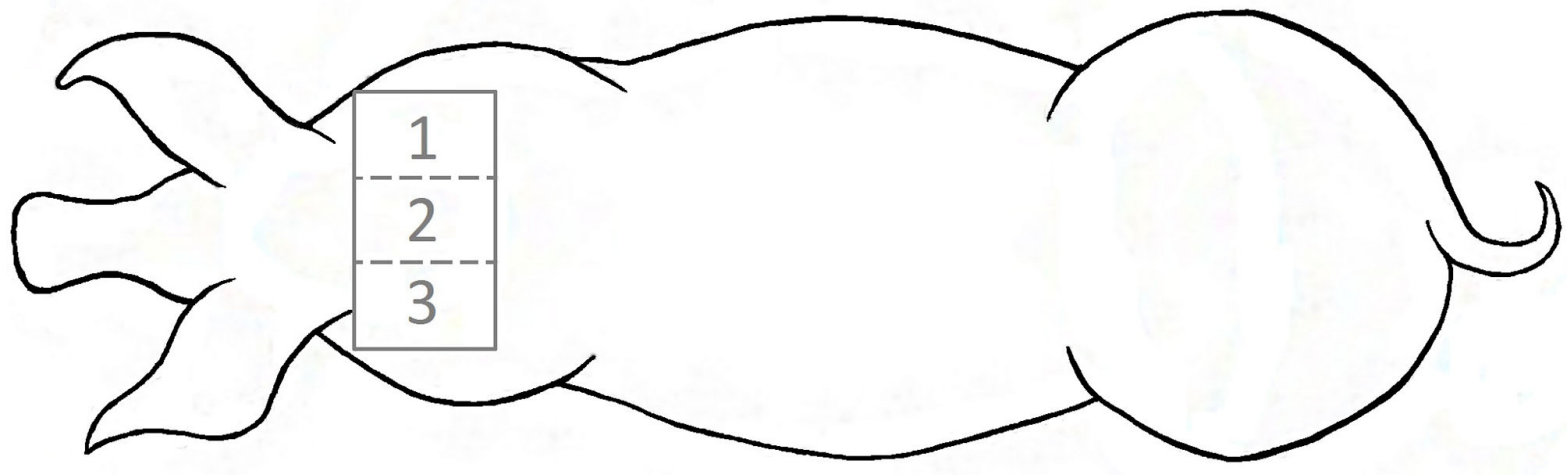
Localization of the shaving area in the transition between neck and shoulder blades with subsections: (1) right lateral part, (2) region over the spine and (3) left lateral part.

Using electronical clippers, a bilateral symmetric area of 20 × 30 cm was shaved in the transition between neck and shoulder blades (Figure 3) as close as possible to the skin. With regard to the multicompartment model (16), this method should rule out, as far as possible, that cortisol in hair originated from outer substances like feces, so as not to falsify the results.

**Figure 4:**
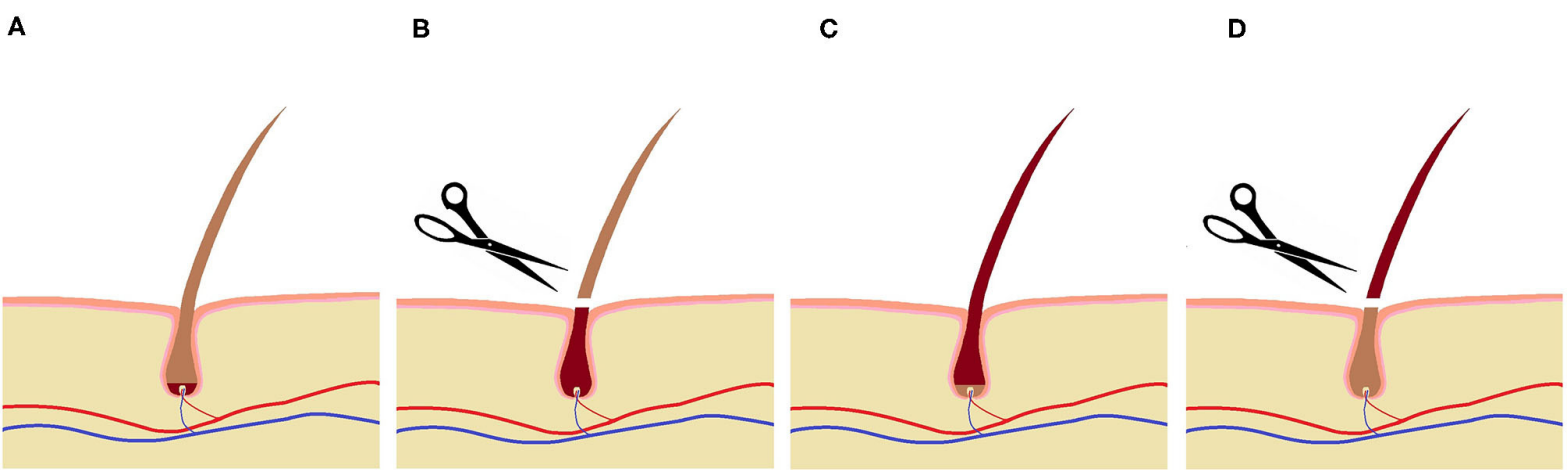
Shaving regime for hair sampling: 
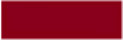
 indicates hair formed in the farrowing systems, 
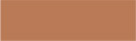
 indicates hair formed outside the farrowing systems; **(A)** at the day of entering the systems, **(B)** 13 days after entering (first shaving), **(C)** at the day of leaving the systems, **(D)** 15 days after leaving (second shave for hair sampling).

In order to take samples from the newly grown hair, representing the period being in the farrowing systems, the surface of interest was shaved twice. Considering a depth of the hair shaft in the skin of 3–4 mm (27) and an assumed growth rate of 0.7 cm/month (28), it takes about 2 weeks until the lower part of the hair shaft has reached the outermost skin layer to be shaved. Therefore, the sows were shaved 13 days (between 13:00 and 17:00) after entering the farrowing system. Consequently, the newly grown hair in this region should have been formed in the farrowing housing period. To ensure that the hair formed during the experimental period had grown out of the skin, animals were shaved 15 days after leaving the farrowing system again, thus 35 days after their first shaving (Figure 4). For measuring the hair length aimed at determining the hair growth rate in the experimental sows (*n* = 42), the shaved area was divided into three equal sections: a left and a right lateral part and the median subsection over the spine (Figure 3). The regrown hairs were first measured in length for the different regions before shaving and then stored in airtight plastic bags under light-protected and dry conditions at room temperature. The hair samples taken from the second shave were sent to the University of Technology, Dresden, Germany for analysis. A total of 61 samples (31 from FC sows and 30 from LH sows) were collected and analyzed. For each section, the length of five hairs per sow was determined with a folding rule after 35 days of growth. Thereafter, the hair growth rate was calculated for a 30-day period in order to compare our own results to those of earlier studies.

### Measurement of Hair Cortisol Concentration

#### Cortisol Extraction

Hair sample washing and the extraction of the hair cortisol based on Davenport's methods (13) were performed. The hair segments were put into a 10 mL glass vessel into which 2.5 mL isopropanol was subsequently added. The tube was then transferred to an overhead rotator, where the prepared samples were mixed for 3 min at room temperature. A short washing time was chosen simply to remove residual traces of externally originating cortisol from the surface of the hair, without extracting steroids from the interior of the hair shaft. The washing procedure was repeated twice and the hair samples were then dried for at least 12 h. A total of 7.5 mg of the washed and dried hair samples was placed in a 2 mL cryo vial for the following 18 h and incubated in 1.5 mL pure methanol for cortisol extraction. The hair samples were then fed into a microcentrifuge and then spun at 10,000 rpm for 2 min. A total of 1 mL of the clear supernatant was transferred to a new 2 mL glass vial. Under a steady stream of nitrogen gas and temperatures of 50 degrees Celsius, alcohol was evaporated and the samples were totally dried. To reconstitute the dried extracts, 0.4 mL of distilled water was added and the tube was vortexed for 15 s.

#### Cortisol Concentration by Immunoassay

The hair cortisol concentration was finally analyzed using a commercially available immunoassay with chemiluminescence detection (CLIA, IBL International GmbH, Hamburg, Germany). Due to low concentrations of cortisol in the hair, the protocol “RE62019” for ultra-sensitive detection was followed. This included the preparation of an additional standard by diluting standard B 1:3 with standard A. One hundred microliters of each standard, control and sample were pipetted into the respective wells of the microtiter plate. The enzyme conjugate was diluted at 75% and 50 microliters of this was added into each well. The plate was then incubated for 3 h at room temperature on an orbital shaker (400–600 rpm). After washing the plate four times with 250 microliters of diluted wash buffer, 50 microliters of prepared substrate solution mixture were pipetted into each well. The measurement of the relative luminescence units was performed after 10 min.

The assay precision in this study, indicated by the intra- (variation within plates) and interassay (variation between plates) coefficient of variance, was below 10 and 12%, respectively.

### Statistical Analysis

Statistical analyses were performed using the R statistics software (29). The level of significance was set at *P* < 0.05.

Data were tested for normal distribution by using histograms.

#### Hair Growth Rate

A linear mixed effects model was used for hair length analysis in different body regions by implementing the R package *Imer Test* (30), with “hair length” as the dependent variable and “body region” as fixed and “sow” as well as “batch” as random effects. To determine any differences in the hair growth rate between the three different body regions, multiple pairwise comparisons of all body regions were carried out using *t*-tests by implementing the R package emmeans (31). The resulting *p*-values were adjusted using the Bonferroni- Holm method.

In the following, hair lengths are stated as means ± standard deviations (SD).

#### Hair Cortisol Concentration

First, the measured cortisol values were logarithmized to approach a normal distribution. A linear model was used with hair cortisol concentration as the dependent variable and the following fixed effects: farrowing system, sows' parity, number of piglets born alive, number of weaned piglets, total piglet loss, mean temperature in the experimental period, sows' weight loss in the farrowing system, occurrence of stereotypies, BLS and ULS on the third day of investigation (Supplementary Table 1).

Prior to the analysis, the potential effects were prioritized according to their potential influence on HCC. Thereafter, they were included stepwise in the model before the final model was developed.

The effects could potentially affect the stress level of sows and were investigated for the following reasons:

1. The housing system to unveil potential environmental effects on stress level
2. Parity, to show the effects of age and life experience on stress level
3. The number of born, weaned, piglet loss to uncover the effects of litter sizes on the sows' stress level
4. Temperature, to illuminate heat or cold stress
5. Weight loss of sows, to discover possible links between the physical conditions and the sows' stress level
6. Stereotypies, to show any link between behavior and stress
7. Lesions scoring results, to determine if body/udder lesions were stress-related.

The effects of the model were examined for significance using *t*-tests.

The stated mean values and standard deviations were calculated from the measured, not the modeled (logarithmized), HCC values.

#### Body Lesion Score

A logarithmic mixed-effects regression model was used in conjunction with the R package *Imer Test* (30) to analyze the BLS, considering the following fixed effects: farrowing system, time of investigation, number of weaned piglets, sows' parity, sows' body weight at the day of entering the systems, sows' weight loss in the farrowing system (Supplementary Table 2). Sow, pen and batch were considered as random effects. Prior to the analysis, the potential effects were prioritized according to their potential influence on HCC. Thereafter, they were included stepwise in the model before the final model was developed.

The effects could potentially affect the lesion score of sows and were chosen for the following reasons:

1. Farrowing system, in order to reveal environmental effects on the lesion score
2. Time of investigation, in order to reveal the effect of the housing period on the lesion score
3. Number of weaned piglets, in order to reveal the effects of litter sizes on lesions
4. Sows' parity, in order to reveal the effects of age and life experience on the lesion score
5. Sows' body weight at the day of entering the systems, in order to reveal the effects of weight/force on the lesion score
6. Sows' weight loss in the farrowing system to show any link between body weight or nutrition and the lesion score.

Based on the final model, *posthoc*-analysis was conducted using *t*-tests and the R package *emmeans* (31) to examine any differences in BLS between the three times of investigation within each housing system and between the two systems at each time of investigation. Using the Bonferroni-Holm method, the *P*-values were adjusted.

## Results

### Hair Growth Rate

Highly significant differences were found in the hair growth rate between different regions of the shaving area. While the hair in both lateral parts of the shaving area grew almost identically in length within 30 days (left side: 7.48 ± 3.52 mm, right side: 7.44 ± 3.24 mm, *P*_adj_ = 1.00), there was considerably more growth in the 10 cm-wide area above the spine (12.27 ± 3.95 mm, *P*_adj_ < 0.0001) (Supplementary Tables 3, 4).

### Hair Cortisol Concentration

**Figure 5:**
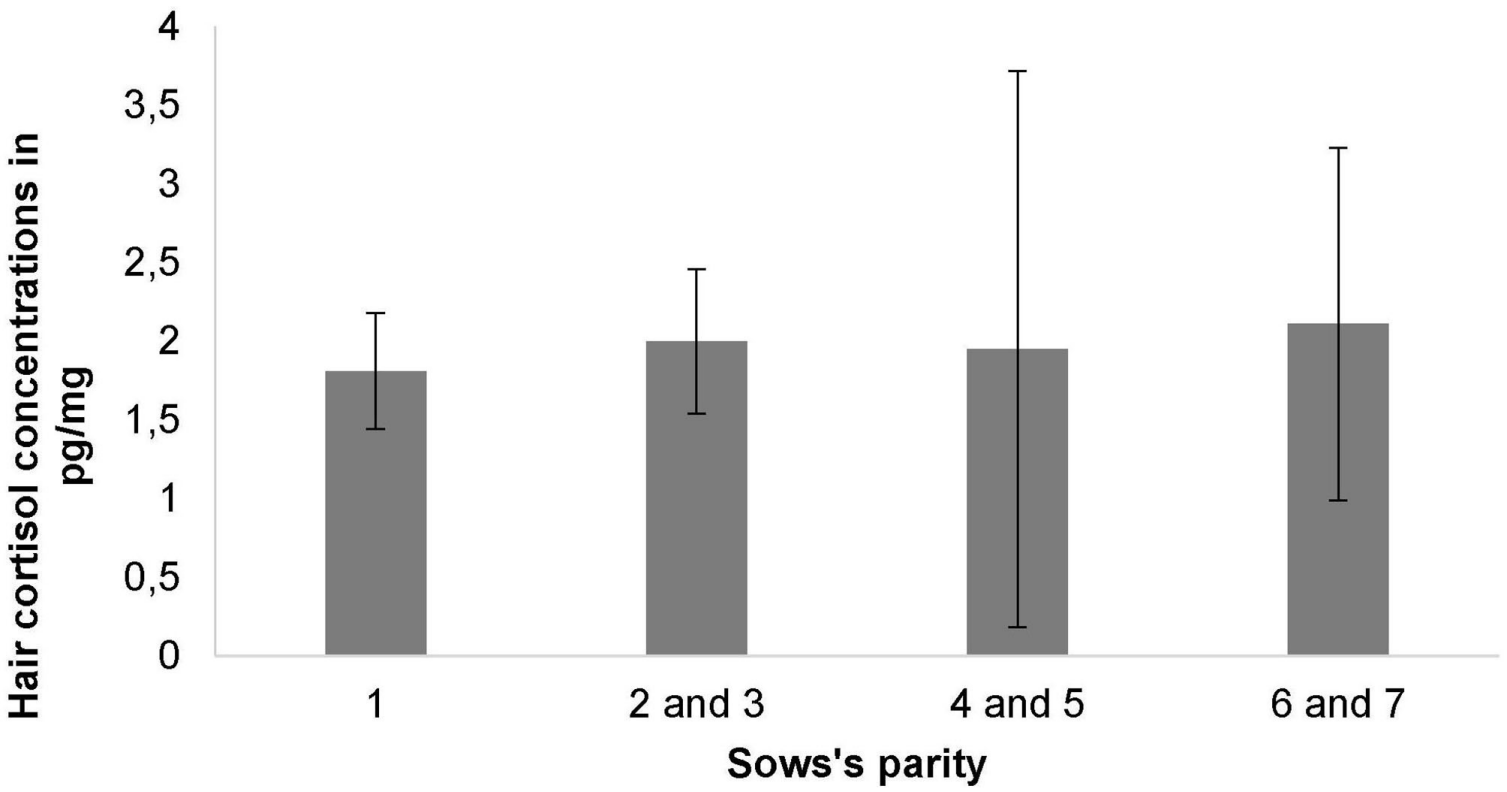
Mean hair cortisol concentrations and standard deviations depending on the sows' parity.

Overall, HCC were measured from a minimum of 0.49 pg/mg to a maximum of 8.92 pg/mg with a mean of 1.99 ± 1.23 pg/mg for all analyzed samples. Mean HCC did not differ significantly between the farrowing systems (LH: 1.85 + 0.82 pg/mg, FC: 2.13 + 1.53 pg/mg, *P* = 0.631) (Table 3). HCC was also not affected by the sows' parity (Figure 5), the number of piglets born alive, the number of weaned piglets, the number of total piglet loss, the skin lesion score, the udder lesion score, individual body weight loss during the study period, the occurrence of stereotypies or climatic conditions in the compartment (*P* > 0.05).

**Table 3:** Descriptive results of hair cortisol concentrations (pg/mg) in the two farrowing systems (LH, loose housing; FC, farrowing crate).

**System**	**N**	**Median**	**Mean**	**SD**	**Min**	**Max**
LH	30	1.735	1.853	0.817	0.490	3.730
FC	31	1.630	2.125	1.526	0.940	8.920

### Skin Lesions

**Figure 6:**
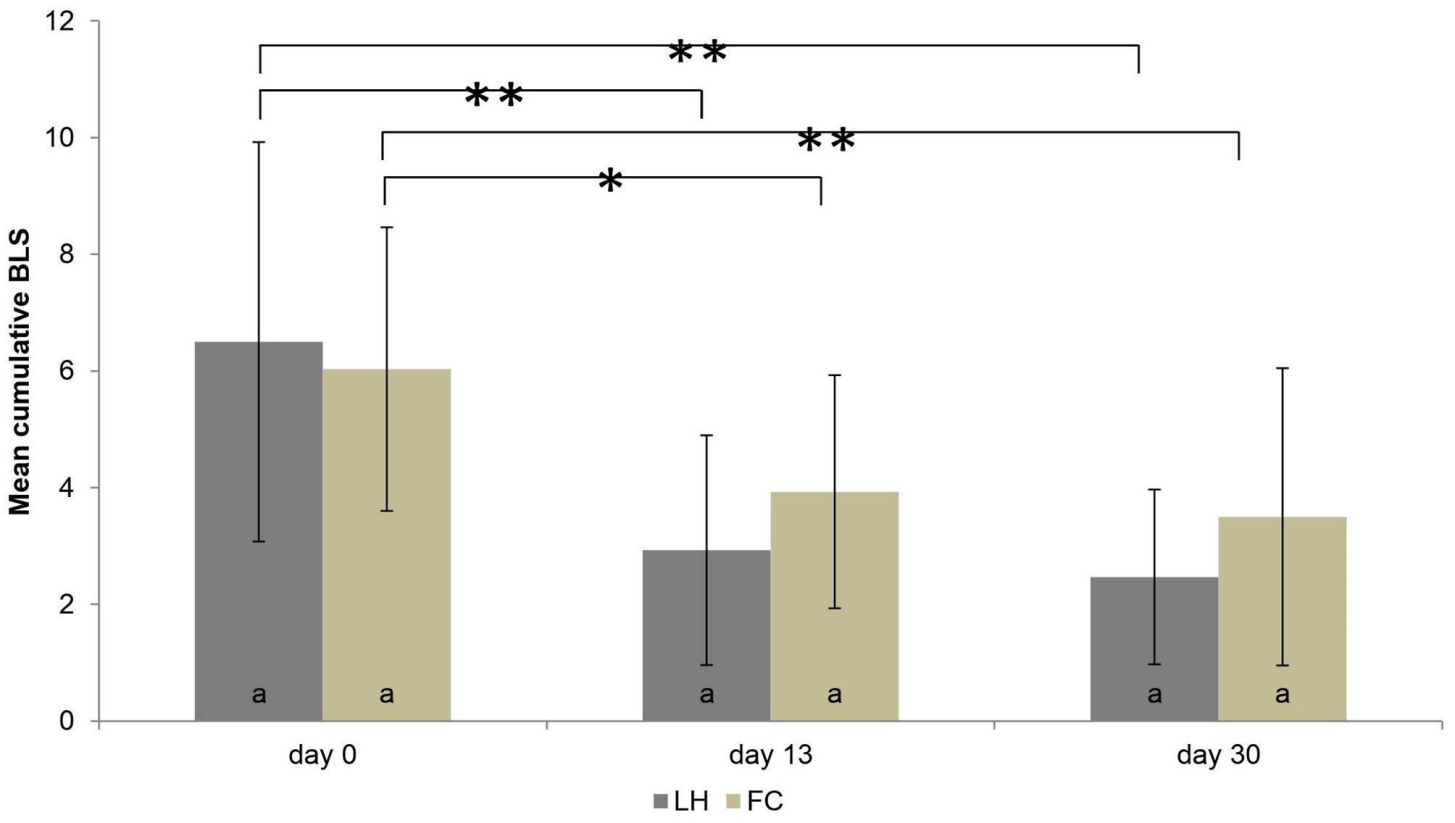
Mean body lesion score (BLS) and standard deviations of the mean in the two systems (pens with farrowing crate = FC, loose housing pens = LH, each *n* = 30 sows) on three examination days. While there were no significant differences within one examination day between the systems (marked by same letters), the significant differences between the examination days are marked by * *P* < 0.05 or ** *P* < 0.001.

In both housing systems, the mean individual BLS declined from the beginning to the end of the housing period (*P* < 0.001) (Figure 6). However, no difference in the mean individual BLS was found between FC sows and LH sows, in general (*P* = 0.895). Also, when analyzing each observation time separately (day 0, day 13, day 30), the mean BLS did not differ significantly between FC and LH sows (*P* > 0.05). The number of weaned piglets, sows' parity, body weight at the day of entering the systems, as well as the weight loss of the sows in the farrowing systems had no influence on the BLS (all *P* > 0.05). The results of the BLS at the three examination times are shown in Figure 6.

## Discussion

### Hair Growth Rate

In order to use HCC as a retrospective calendar of stress, knowledge about the speed of hair growth is important. Only then can the period reflected by HCC be narrowed down, as this is how the sampling interval is determined (19). Furthermore, varying hair growth rates between different body regions can cause different results in HCC of these samples within the same examination period (32). Consequently, measurements of the hair growth rate are indispensable when evaluating hair cortisol as an indicator for chronic stress during a specific time frame. However, several studies on HCC in pigs did not consider the hair growth rate in order to adapt the shaving scheme to the period under investigation, or they did not perform a first shave (28, 32). In studies with a non-specific time frame for stress detection (25, 33, 34), prior shaving of the sampling region seems unnecessary. If the stress level should be studied over a certain period of time, shaving should be carried out beforehand.

In human studies, a mean hair growth rate of scalp hair of 1 cm/month is generally accepted. Nonetheless, scalp hair growth varies according to the region, with the posterior vertex region accepted to be the one with the most uniform growth rates, resulting in less intra-individual variation of HCC. Thus, samples are typically obtained from this region (12, 23, 35). However, such a standardized procedure does not yet exist for hair sampling in pigs and it seems useful to identify most suitable body regions for hair sampling in these animals. It was already shown in pigs that the HCC of the neck was lower than that of the lumbar region (32), which in turn was lower than that of the tail (34). Hair growth rates in these regions partly differed significantly, with the lowest one in the neck (34). To the best of our knowledge, there has not yet been any study performed on pigs measuring the growth rate of hair in different subareas within the same or adjacent body regions. In the present study, hair growth rate in the lateral areas of the shaving region was almost identical to those found by Bacci et al. (28) in the rump region of sows and similar to those found by Heimbürge et al. (34) in the neck. However, the growth rate determined in the present study for the subarea above the spine differed from that found in the lateral areas within the same shaving region. Thus, while Heimbürge et al. (34) revealed differences in the hair growth rate between different body regions in pigs, the present study also showed evidence that such differences were present within a single body region in pigs. This important result potentially influencing HCC measurements should be considered in further studies.

### Hair Cortisol Concentration

It is assumed that chronic stress can cause both increased and decreased reaction of the HPA axis at different time points during a stressful situation: an HPA activation and elevated cortisol levels at the beginning can be followed by a counter-regulatory response over time, with rebounded glucocorticoid levels below normal ones by feedback mechanisms (36). The adaptation to recurrent or persistent so-called homotypic stressors in terms of a regulation of the HPA-axis, with a reduced physiological response compared to acute stress experiences, has been known for decades and is referred to as “habituation.” It depends on characteristics of stress exposure, such as severity, modality and duration (37), and is stressor-specific (38). However, the HPA axis does not get used to particularly threatening stressors (39). Confining sows to crates could be seen as a cause of chronic stress. Even if housing-related hypercortisolemia may be transient (40), hair cortisol is recommended in earlier studies as a good indicator of chronic stress.

In the current paper, we explore if methods adapted from previous successful hair cortisol extraction studies (28, 32, 34) also apply to sows in farrowing systems housed under different farrowing conditions by trying to identify different possible and specific stress factors in the farrowing units.

To the best of our knowledge, this is the first study using hair cortisol analyses for evaluating different farrowing systems for sows.

In addition, it was investigated whether the housing systems had physical impacts on the sows using a lesion score, and whether these were related to measured HCC.

Further data obtained in this study concerning animal behavior and performance will be presented in another paper.

However, the results of the present study do not reveal any effects of either the housing system (farrowing crate or loose housing) or of all other investigated parameters on hair cortisol levels of sows. Thus, the results of the present study seem to be partly contradictory to those obtained by previous studies. Trevisan et al. (41) showed an influence of body weight on HCC, with lighter sows having higher hair cortisol concentrations, which was not confirmed in the present study. However, the cross-breeding sows (local genetic × large white) used in this previous study differed considerably in age, mean body weight and hair growth characteristics from the sows used in our study (1.6 ± 0.2 vs. 0.7 ± 0.3 cm/month in the lateral subareas of the shaving field), thus comparability may be limited. Bacci et al. (28) found an influence of the season on hair cortisol concentration in sows, with the lowest concentrations during the hot season. It was shown by Muns et al. (42) that an increase in temperature from 20 to 25°C can induce heat stress in sows. However, such an influence was neither confirmed by Heimbürge et al. (34) nor by the present study, even if mean room temperatures were closely related to the season (Supplementary Table 5) and were quite similar to those measured in the study of Bacci et al. (28) with 20–27°C.

The sampling procedure could also be a cause of conflicting results in different studies. The hair sampling method in the current study was an attempt to improve over previous methods by considering thoroughly the hair growth (which was included for analysis). Due to a standardized procedure in the present study, it is assumed that meaningful results were achieved. In the present study, care was taken to ensure that only hair that was newly formed during the study period was used for hair cortisol measurement. Thus, it was necessary to consider the time delay between incorporating cortisol in the hair and the appearance of this hair section on the skin's surface (24). Therefore, the hair growth rate was determined and the selected body area was shaved when the study started. As this procedure was not performed in all previous studies and in the case of Bacci et al. (28), hair samples were collected alternately from different body sides, the comparability of the results may be limited. Regardless of the selected body site and region for hair sampling, it is important to select the same site for all subjects (14). Since Casal et al. (32) found lower HCC in the dorsal neck of pigs compared to other body regions, suggesting that this lower cortisol concentration may result from less soiling with feces in this body part, this region was also chosen for sampling in the current examination.

Bacci et al. (28) reported that sows had higher hair cortisol values when kept in crates compared to group housing in the gestation area. However, the housing systems changed with the stage of pregnancy, and high cortisol levels in crated sows may also be explained by the systemic rise in cortisol concentration during farrowing (43). In contrast, the present study compared the hair cortisol levels of sows in different housing systems during the same stage of the reproductive cycle. Thus, the effect of the housing system may be better determined. In this case, neither the housing system nor body weight nor other investigated factors affected hair cortisol levels in sows.

Most earlier studies dealing with stress levels of crated and loose-housed sows in farrowing systems used cortisol measurement in saliva or blood and are, therefore, not directly comparable to this study (43–48). Lawrence et al. (43) found higher plasma cortisol values around farrowing independent of the housing system. However, higher cortisol levels were detected in crated sows compared to loose-housed sows during this time. The cortisol levels then decreased rapidly, and were almost identical in sows of both groups only 1 day after farrowing (43).

Hair cortisol is supposed to be an indicator of the previous weeks' or months' adrenocortical activity (14), representing the accumulated hormonal production of the period of hair growth (12). In our study, a fairly long period of investigation after farrowing may have “diluted” higher cortisol levels around farrowing, if followed by lower cortisol values thereafter. Consequently, differences in stress levels between the two housing systems in our study may no longer be obvious when measuring cortisol in hair.

Furthermore, the present study did not reveal any other influencing factors on hair cortisol levels in sows, such as the number of suckling or weaned piglets. This could be explained by the habituation to the constant stressors during the housing period. Suckling was proven to be a stress factor for sows as higher cortisol plasma levels were found the day before weaning compared to the day after weaning. Shortly after weaning, an increase in plasma cortisol was also measured (49). While the weaning procedure is short-lived, the longer suckling period may have a greater impact on cortisol levels. Apart from the possibility that the stimuli were not stressful enough to cause elevated cortisol levels in the sows (50), there is also the possibility of downregulation of the HPA axis in this case. It is also conceivable that dominant stressors with greater influence on cortisol levels mask the impact of other stressors of lesser effect on the HPA axis. An influence of these other stressors (for instance, the housing system) may, therefore, no longer be represented by the measured cortisol values.

Finally, it may also be assumed that measuring hair cortisol in pigs is not the appropriate method to determine stress levels. Heimbürge et al. (51) found no differences in HCC between pigs previously treated with ACTH and control animals, whereas in cattle an effect of ACTH treatment on hair cortisol level was found. The authors concluded that this may be related to a lower systemic cortisol response in pigs, although seasonally lower hair growth or external cross-contamination of hair cannot be ruled out either.

### Skin Lesions

As lesion scores are an important animal welfare indicator, they were further investigated.

Although the farrowing crate itself may cause injuries to the sows (52), this was not reflected by the scoring results of the present study, with no indication of any influence of the farrowing system on the BLS. As shown in other studies investigating farrowing housing systems (26, 53), a significant decrease in BLS was observed in both housing systems during the study period. Individually housing sows in the farrowing systems prevented agonistic behavior, as this occurred in the gestation period when sows were group-housed. Thus, over time, skin lesions resulting from previous group housing during pregnancy healed. From this point of view, the time spent in the farrowing systems can be regarded as a recovery phase. Consequently, at no point in time was HCC found to be influenced by BLS. Also, Carrol et al. (25) did not find any influence of skin lesions on HCC in fattening pigs, whereas HCC was affected by tail lesions and lameness.

## Conclusions

The results of the present study revealed that the use of hair cortisol measurements in sows around farrowing seems to be limited. This may be due to the constant stressful conditions in farrowing systems, such as suckling bouts or single housing, to which the sows' HPA axis may adapt over time, resulting in decreased cortisol levels. However, it is also possible that measuring hair cortisol is not the appropriate method for determining stress in pigs. Thus, a meaningful use of HCC in sows for comparing the effect of different farrowing systems on animal welfare remains questionable. Further research on the time course of cortisol levels mapped in the HCC seems necessary to validate the measured values. Also, the hair growth rate should be considered in further studies when measuring HCC. Only if the collected hair grows more or less homogeneously can the time period of hair analysis be defined. Regional differences in hair growth rate within the same shaving area should, therefore, be considered in future studies.

## Data Availability Statement

The raw data supporting the conclusions of this article will be made available by the authors, without undue reservation.

## Ethics Statement

The animal study was reviewed and approved by Animal Welfare Officer of the University of Veterinary Medicine Hannover, Hannover, Foundation, Germany. Written informed consent was obtained from the owners for the participation of their animals in this study.

## Author Contributions

D-HW and MF designed the experiments. D-HW collected the data. SB and SH analyzed the data and performed the statistical analysis. D-HW, MF, NK, SB, and SH wrote and revised the paper. All authors contributed to the article and approved the submitted version.

## Conflict of Interest

The authors declare that the research was conducted in the absence of any commercial or financial relationships that could be construed as a potential conflict of interest.

## References

[1] Lassen J Sandøe P Forkman B Happy pigs are dirty! – conflicting perspectives on animal welfare. Livestock Sci. (2006) 103:221–30. 10.1016/j.livsci.2006.05.008

[2] EFSA (European Food Safety Authority) Scientific opinion of the panel on animal health and welfare on a request from the commission on animal health and welfare aspects of different housing and husbandry systems for adult breeding boars, pregnant, farrowing sows and unweaned piglets. EFSA J. (2007) 572:1–13. 10.2903/j.efsa.2007.572

[3] Pedersen LJ Malmkvist J Andersen HML Housing of sows during farrowing: a review on pen design, welfare and productivity. In: Aland A, Banhazi T, editors. Livestock Housing: Modern Management to Ensure Optimal Health and Welfare of Farm Animals. Wageningen Academic Publishers (2013). p. 93–111. 10.3920/978-90-8686-771-4_05

[4] EFSA (European Food Safety Authority) EFSA panel on animal health and welfare (AHAW); scientific opinion on the use of animal-based measures to assess welfare in pigs. EFSA J. (2012) 10:2512 10.2903/j.efsa.2012.2512 PMC13092913 42016070

[5] Mkwanazi MV Ncobela CN Kanengoni AT Chimonyo M. Effects of environmental enrichment on behaviour, physiology and performance of pigs - A review. Asian Austr J Anim Sci. (2019) 32:1–13. 10.5713/ajas.17.0138 28728387 PMC6325398

[6] FAWC (Farm Animal Welfare Council) Farm Animal Welfare in Great Britain: Past, Present and Future. (2009). Available online at: https://www.gov.uk/government/publications/fawc-report-on-farm-animal-welfare-in-great-britain-past-present-and-future (accessed May 26, 2020).

[7] Veissier I Boissy A. Stress and welfare: two complementary concepts that are intrinsically related to the animal's point of view. Physiol Behav. (2007) 92:429–33. 10.1016/j.physbeh.2006.11.008 17182067

[8] Matteri RL Carroll JA Dyer CJ Neuroendocrine responses to stress. In: Moberg GP, Mench JA, editors. The Biology of Animal Stress: Basic Principles and Implications for Animal Welfare. Wallingford: CABI Publishing (2000).

[9] Mormède P Andanson S Aupérin B Beerda B Guémené D Malmkvist J. Exploration of the hypothalamic-pituitary-adrenal function as a tool to evaluate animal welfare. Physiol Behav. (2007) 92:317–39. 10.1016/j.physbeh.2006.12.003 17234221

[10] Martínez-Miró S Tecles F Ramón M Escribano D Hernández F Madrid J. Causes, consequences and biomarkers of stress in swine: an update. BMC Vet Res. (2016) 12:171. 10.1186/s12917-016-0791-8 27543093 PMC4992232

[11] Herman JP. Neural control of chronic stress adaption. Front Behav Neurosci. (2013) 7:61. 10.3389/fnbeh.2013.00061 23964212 PMC3737713

[12] Russell E Koren G Rieder M Van Uum S. Hair cortisol as a biological marker of chronic stress: current status, future directions and unanswered questions. Psychoneuroendocrinology. (2012) 37:589–601. 10.1016/j.psyneuen.2011.09.009 21974976

[13] Davenport MD Tiefenbacher S Lutz CK Novak MA Meyer JS. Analysis of endogenous cortisol concentrations in the hair of rhesus macaques. Gen Comp Endocrinol. (2006) 147:255–61. 10.1016/j.ygcen.2006.01.005 16483573

[14] Meyer JS Novak MA. Minireview: hair cortisol: a novel biomarker of hypothalamic-pituitary-adrenocortical activity. Endocrinology. (2012) 153:4120–7. 10.1210/en.2012-1226 22778226 PMC3423616

[15] Koren L Mokady O Karaskov T Klein J Koren G Geffen E A novel method using hair for determining hormonal levels in wildlife. Anim Behav. (2002) 63:403–6. 10.1006/anbe.2001.1907

[16] Henderson GL. Mechanisms of drug incorporation into hair. Forensic Sci Int. (1993) 63:19–29. 10.1016/0379-0738(93)90256-A 8138221

[17] Ito N Ito T Kromminga A Bettermann A Takigawa M Kees F. Human hair follicles display a functional equivalent of the hypothalamic-pituitary-adrenal axis and synthesize cortisol. FASEB J. (2005) 19:1332–4. 10.1096/fj.04-1968fje 15946990

[18] Sharpley CF Kauter KG McFarlane JR. An initial exploration of *in vivo* hair cortisol responses to a brief pain stressor: latency, localization and independence effects. Physiol Res. (2009) 58:757–61. 19093721 10.33549/physiolres.931544

[19] Kirschbaum C Tietze A Skoluda N Dettenborn L. Hair as a retrospective calendar of cortisol production-increased cortisol incorporation into hair in the third trimester of pregnancy. Psychoneuroendocrinology. (2009) 34:32–7. 10.1016/j.psyneuen.2008.08.024 18947933

[20] Thomson S Koren G Fraser LA Rieder M Friedman TC Van Uum SH. Hair analysis provides a historical record of cortisol levels in Cushing's syndrome. Exp Clin Endocrinol Diabetes. (2010) 118:133–8. 10.1055/s-0029-1220771 19609841 PMC2945912

[21] González-de-la-Vara Mdel R Valdez RA Lemus-Ramirez V Vázquez-Chagoyán JC Villa-Godoy A Romano MC. Effects of adrenocorticotropic hormone challenge and age on hair cortisol concentrations in dairy cattle. Can J Vet Res. (2011) 75:216–21. 22210998 PMC3122973

[22] Accorsi PA Carloni E Valsecchi P Viggiani R Gamberoni M Tamanini C. Cortisol determination in hair and faeces from domestic cats and dogs. Gen Comp Endocrinol. (2008) 155:398–402. 10.1016/j.ygcen.2007.07.002 17727851

[23] Sauvé B Koren G Walsh G Tokmakejian S Van Uum SH Measurement of cortisol in human hair as a biomarker of systemic exposure. Clin Invest Med. (2007) 30:E183–91. 10.25011/cim.v30i5.2894 17892760

[24] Heimbürge S Kanitz E Otten W. The use of hair cortisol for the assessment of stress in animals. Gen Comp Endocrinol. (2019) 270:10–7. 10.1016/j.ygcen.2018.09.016 30287191

[25] Carroll GA Boyle LA Hanlon A Palmer MA Collins L Griffin K. Identifying physiological measures of lifetime welfare status in pigs: exploring the usefulness of haptoglobin, C- reactive protein and hair cortisol sampled at the time of slaughter. Ir Vet J. (2018) 71:8. 10.1186/s13620-018-0118-0 29507716 PMC5833096

[26] Nicolaisen T Risch B Lühken E van Meegen C Fels M Kemper N. Comparison of three different farrowing systems: skin lesions and behaviour of sows with special regard to nursing behaviour in a group housing system for lactating sows. Animal. (2019) 13:2612–20. 10.1017/S1751731119000661 31104635 PMC6801640

[27] Mowafy M Cassens RG Hair growth in the domestic pig-histological aspects. J Am Leather Chem Assoc. (1976) 71:64–70.

[28] Bacci ML Nannoni E Govoni N Scorrano F Zannoni A Forni M. Hair cortisol determination in sows in two consecutive reproductive cycles. Reprod Biol. (2014) 14:218–23. 10.1016/j.repbio.2014.06.001 25152520

[29] R Core Team R: A Language and Environment for Statistical Computing. R Foundation for Statistical Computing (2019). Available online at: https://www.R-project.org/ (accessed May 30, 2020).

[30] Kuznetsova A Brockhoff PB Christensen RHB lmerTest package: tests in linear mixed effects models. J Stat Softw. (2017) 82:13 10.18637/jss.v082.i13

[31] Lenth R emmeans: Estimated Marginal Means, aka Least-Squares Means. R package version 1.4.3.01. (2019). Available online at: https://CRAN.R-project.org/package=emmeans (accessed May 30, 2020).

[32] Casal N Manteca X Peña LR Bassols A Fàbrega E Analysis of cortisol in hair samples as an indicator of stress in pigs. J Vet Behav. (2017) 19:1–6. 10.1016/j.jveb.2017.01.002

[33] Bergamin C Comin A Corazzin M Faustini M Peric T Scollo A. Cortisol, DHEA, and sexual steroid concentrations in fattening pigs' hair. Animals. (2019) 9:345. 10.3390/ani9060345 31212851 PMC6616490

[34] Heimbürge S Kanitz E Tuchscherer A Otten W. Within a hair's breadth - factors influencing hair cortisol levels in pigs and cattle. Gen Comp Endocrinol. (2020) 288:113359. 10.1016/j.ygcen.2019.113359 31830475

[35] Pragst F Balikova MA. State of the art in hair analysis for detection of drug and alcohol abuse. Clin Chim Acta. (2006) 370:17–49. 10.1016/j.cca.2006.02.019 16624267

[36] Miller GE Chen E Zhou ES. If it goes up, must it come down? Chronic stress and the hypothalamic-pituitary-adrenocortical axis in humans. Psychol Bull. (2007) 133:25–45. 10.1037/0033-2909.133.1.25 17201569

[37] Grissom N Bhatnagar S. Habituation to repeated stress: get used to it. Neurobiol Learn Mem. (2009) 92:215–24. 10.1016/j.nlm.2008.07.001 18667167 PMC2773683

[38] Jean Kant G Eggleston T Landman-Roberts L Kenion CC Driver GC Meyerhoff JL. Habituation to repeated stress is stressor specific. Pharmacol Biochem Behav. (1985) 22:631–4. 10.1016/0091-3057(85)90286-2 2986182

[39] Figueiredo HF Bodie BL Tauchi M Dolgas CM Herman JP. Stress integration after acute and chronic predator stress: differential activation of central stress circuitry and sensitization of the hypothalamo-pituitary-adrenocortical axis. Endocrinology. (2003) 144:5249–58. 10.1210/en.2003-0713 12960031

[40] Janssens CJ Helmond FA Wiegant VM. The effect of chronic stress on plasma cortisol concentrations in cyclic female pigs depends on the time of day. Domest Anim Endocrinol. (1995) 12:167–77. 10.1016/0739-7240(94)00018-V 7600767

[41] Trevisan C Montillo M Prandi A Mkupasi EM Ngowi HA Johansen MV. Hair cortisol and dehydroepiandrosterone concentrations in naturally Taenia solium infected pigs in Tanzania. Gen Comp Endocrinol. (2017) 246:23–8. 10.1016/j.ygcen.2017.03.007 28322765 PMC5396532

[42] Muns R Malmkvist J Larsen MLV Sørensen D Pedersen LJ. High environmental temperatures around farrowing induced heat stress in crated sows. J Anim Sci. (2016) 94:377–84. 10.2527/jas.2015-9623 26812342

[43] Lawrence AB Petherick JC McLean KA Deans LA Chirnside J Gaughan A The effect of environment on behaviour, plasma cortisol and prolactin in parturient sows. Appl Anim Behav Sci. (1994) 39:313–30. 10.1016/0168-1591(94)90165-1

[44] Biensen NJ von Borell EH Ford SP. Effects of space allocation and temperature on periparturient maternal behaviors, steroid concentrations, and piglet growth rates. J Anim Sci. (1996) 74:2641–8. 10.2527/1996.74112641x 8923178

[45] Jarvis S Calvert SK Stevenson J van Leeuwen N Lawrence AB Pituitary-adrenal activation in pre-parturient pigs (Sus scrofa) is associated with behavioural restriction due to lack of space rather than nesting substrate. Anim Welfare. (2002) 11:371–84.

[46] Oliviero C Heinonen M Valros A Hälli O Peltoniemi OA. Effect of the environment on the physiology of the sow during late pregnancy, farrowing and early lactation. Anim Reprod Sci. (2008) 105:365–77. 10.1016/j.anireprosci.2007.03.015 17449206

[47] Hales J Moustsen VA Nielsen MBF Hansen CF The effect of temporary confinement of hyperprolific sows in Sow Welfare and Piglet protection pens on sow behaviour and salivary cortisol concentrations. Appl Anim Behav Sci. (2016) 183:19–27. 10.1016/j.applanim.2016.07.008

[48] Goumon S Leszkowová I Šimecková M Illmann G. Sow stress levels and behavior and piglet performances in farrowing crates and farrowing pens with temporary crating. J Anim Sci. (2018) 96:4571–8. 10.1093/jas/sky324 30102369 PMC6247827

[49] Tsuma VT Einarsson S Madej A Lundeheim N Cortisol and β-endorphin levels in peripheral circulation around weaning in primiparous sows. Anim Reprod Sci. (1995) 37:175–82. 10.1016/0378-4320(94)01330-O

[50] Schmitt O Baxter EM Boyle LA O'Driscoll K. Nurse sow strategies in the domestic pig: I. Consequences for selected measures of sow welfare. Animal. (2019) 13:580–9. 10.1017/S175173111800160X 29986790

[51] Heimbürge S Kanitz E Tuchscherer A Otten W Is it getting in the hair? - Cortisol concentrations in native, regrown and segmented hairs of cattle and pigs after repeated ACTH administrations. Gen Comp Endocrinol. (2020) 295:113534 10.1016/j.ygcen.2020.113534 32540492

[52] Anil L Anil SS Deen J. Evaluation of the relationship between injuries and size of gestation stalls relative to size of sows. J Am Vet Med Assoc. (2002) 221:834–6. 10.2460/javma.2002.221.834 12322922

[53] Schrey L Kemper N Fels M Behaviour and skin injuries of sows kept in a novel group housing system during lactation. J Appl Anim Res. (2018) 46:749–57. 10.1080/09712119.2017.1394308

