# Supplementary material for: Analysis of Hair Cortisol as an Indicator of Chronic Stress in Pigs in Two Different Farrowing Systems
Source: Front Vet Sci. 2021 Jan 28;8:605078. doi: 10.3389/fvets.2021.605078 (PMC7876061; doi:10.3389/fvets.2021.605078)
Supplement: Supplementary file 1 [file Data_Sheet_1.docx]

***Supplementary Material***

**Supplementary Tables**

**Supplementary Table 1.** Variables of hair cortisol concentration analysis in the linear mixed effects model .

| Variable | Variable type | Range | Level |
| --- | --- | --- | --- |
| Farrowing system | categorical | - | LH (n=30) / FC (n=31) |
| Parity | continuous | 1 -7 | - |
| Live-born piglets | continuous | 9 - 23 | - |
| Weaned piglets | continuous | 5 - 14 | - |
| Total piglet loss | continuous | 0 -17 | - |
| Temperature | continuous | 20.4°C – 25.2°C | - |
| Weight loss | continuous | 0.04 – 0.23 | - |
| Occurrence of stereotypes | categorical | - | yes (n=20) / no (n=32) |
| BLS, day 30 | continuous | 0 - 9 | - |
| ULS, day 30 | continuous | 0 -4 | - |

**Supplementary Table 2.** Variables of body lesion score analysis in the linear mixed effects model .

| Variable | Variable type | Range | Level |
| --- | --- | --- | --- |
| Farrowing system | categorical | - | LH (n=30) / FC (n=30) |
| Time of investigation | categorical | - | day 0 / 13 / 30 (all n=60) |
| Parity | continuous | 1 -7 | - |
| Weaned piglets | continuous | 5 - 14 | - |
| Body weight | continuous | 233.5kg – 371.1kg | - |
| Weight loss | continuous | 0.036 – 0.228 | - |

**Supplementary Table 3.** Descriptive results of hair length measurements (30-day period), given in mm for the respective number of hairs (N).

| Shaving area | N | Median | Mean | SD | Min | Max |
| --- | --- | --- | --- | --- | --- | --- |
| Left | 210 | 7.71 | 7.48 | 3.52 | 1.71 | 17.14 |
| Spine | 210 | 12.86 | 12.27 | 3.95 | 1.71 | 25.71 |
| Right | 210 | 7.71 | 7.44 | 3.24 | 1.71 | 15.43 |
| Left + right | 420 | 7.71 | 7.46 | 3.38 | 1.71 | 17.14 |

**Supplementary Table 4.** Results of hair length analysis from the linear mixed effects model with pairwise comparisons of body regions.

| Contrast | Estimate | SE | DF | T-ratio | P-value | Adj. P-value |
| --- | --- | --- | --- | --- | --- | --- |
| Left - median | -0.479 | 0.031 | 1010.15 | -15.341 | <10^-16^ | <10^-16^ |
| Left - right | 0.004 | 0.031 | 1010.15 | 0.131 | 0.896 | 1 |
| Left - lateral | 0.002 | 0.027 | 1010.15 | 0.075 | 0.940 | 1 |
| Median - right | 0.483 | 0.031 | 1010.15 | 15.471 | <10^-16^ | <10^-16^ |
| Median - lateral | 0.481 | 0.027 | 1010.15 | 17.789 | <10^-16^ | <10^-16^ |
| Right - lateral | -0.002 | 0.027 | 1010.15 | -0.075 | 0.940 | 1 |

**Supplementary Table 5.** Temperatures (in °C) of the consecutive batches in the two farrowing systems (loose-housing pens – LH, pens with farrowing crate – FC).

| System | Batch | Month | Mean | SD | Min | Max |
| --- | --- | --- | --- | --- | --- | --- |
| LH | 1 | June / July | 22.72 | 1.51 | 18.74 | 27.18 |
|  | 2 | July / Aug | 24.57 | 2.97 | 18.39 | 34.38 |
|  | 3 | Aug / Sep | 22.36 | 1.79 | 18.28 | 29.14 |
|  | 4 | Oct / Nov | 21.32 | 1.08 | 17.76 | 27.52 |
|  | 5 | Nov / Dec | 20.51 | 0.26 | 18.87 | 21.50 |
|  | 6 | Dec / Jan | 20.41 | 0.36 | 16.27 | 21.60 |
| FC | 1 | June / July | 23.53 | 1.03 | 21.70 | 27.11 |
|  | 2 | July / Aug | 25.16 | 2.71 | 21.43 | 35.44 |
|  | 3 | Aug / Sep | 23.62 | 1.31 | 21.70 | 30.23 |
|  | 4 | Oct / Nov | 22.05 | 1.00 | 20.45 | 28.07 |
|  | 5 | Nov / Dec | 21.13 | 0.72 | 20.04 | 22.81 |
|  | 6 | Dec / Jan | 21.00 | 0.71 | 19.89 | 22.66 |
